# Tellimagrandin II, A Type of Plant Polyphenol Extracted from *Trapa bispinosa* Inhibits Antibiotic Resistance of Drug-Resistant *Staphylococcus aureus*

**DOI:** 10.3390/ijms20225790

**Published:** 2019-11-18

**Authors:** Yu-Wei Chang, Wan-Chun Huang, Chun-Yu Lin, Wen-Hung Wang, Ling-Chien Hung, Yen-Hsu Chen

**Affiliations:** 1 Graduate Institute of Medicine, College of Medicine, Kaohsiung Medical University, Kaohsiung 80708, Taiwan; golden3p@gmail.com (Y.-W.C.); infectionman@gmail.com (C.-Y.L.); 2 Department of Laboratory, Taitung Hospital, Ministry of Health and Welfare, Taitung 95043, Taiwan; 3 School of Medicine, Graduate Institute of Medicine, Sepsis Research Center, Center of Tropical Medicine and Infectious diseases, Kaohsiung Medical University, Kaohsiung 80708, Taiwan; echoeshuang@gmail.com (W.-C.H.); bole0918@gmail.com (W.-H.W.); lavender99kimo@yahoo.com.tw (L.-C.H.); 4 Division of Infectious Disease, Department of Internal Medicine, Kaohsiung Medical University Hospital, Kaohsiung Medical University, Kaohsiung 80756, Taiwan; 5 Department of Internal Medicine, Kaohsiung Municipal Ta-Tung Hospital, Kaohsiung 80145, Taiwan; 6 Department of Biological Science and Technology, College of Biological Science and Technology, National Chiao Tung University, HsinChu 30010, Taiwan

**Keywords:** MRSA, TGII, synergistic effect, combination therapy

## Abstract

The emergence of methicillin-resistant *Staphylococcus aureus* (MRSA) has become a critical global concern. Identifying new candidates of anti-*S. aureus* agents is urgently required because the therapeutic strategies for infected patients are limited currently. Therefore, the present study investigated whether Tellimagrandin II (TGII), a pure compound extracted from the shells of *Trapa bispinosa*, exhibits antibacterial effects against MRSA. We first showed that TGII exerted potent inhibitory activity against MRSA with a minimum inhibitory concentration of 128 μg/mL. The obtained fractional inhibitory concentration suggested that TGII could alone exert antistaphylococcal activity, and TGII combined with low doses of antibiotics displayed synergistic effects against MRSA. Moreover, we found that TGII exerted bactericidal activity by reducing the expression of *mecA* followed by the negative regulation of the penicillin-binding protein 2a (PBP2a) of MRSA. Transmission electron microscopy (TEM) images further confirmed that TGII destroyed the integrity of the cell wall of MRSA and caused the loss of cytoplasm content. In conclusion, we evidenced the antibacterial effects of TGII against MRSA, which enables the effective dose of current antibiotics to be reduced and the predicament of drug-resistant *S. aureus* isolates to be overcome.

## 1. Introduction

*Staphylococcus aureus*, a gram-positive bacterium belonging to the *Staphylococcaceae* family, is the leading cause of infectious outbreaks, including complicated wounds, food poisoning, and nosocomial infections [1,2]. It is an important pathogen in both healthcare and community settings, and it causes opportunistic infections when the patient’s immune defense is compromised due to immune deficiency caused by catheter or ventilator use or surgery. [3,4]. Notably, bacteremia caused by *S. aureus* is a frequent concern in hospital-associated infections and accounts for approximately 20% of all cases of bloodstream infectious diseases, mainly resulting in high morbidity and mortality [5]. Furthermore, epidemiological studies have highlighted that both methicillin-sensitive *S. aureus* (MSSA) and methicillin-resistant *S. aureus* (MRSA) cause high mortality, but outcomes from MRSA are worse than those from MSSA with an overall mortality of 20–50% [5,6].

Tellimagrandin II (TGII) is a natural compound with wide medicinal use worldwide. It is a type of plant polyphenol extracted from the shells of *Trapa bispinosa* [7]. In plants, polyphenols serve as secondary metabolites and play a critical role in defense against external stress, including ultraviolet radiation and pathogen infection [8]. In the past decade, polyphenolic compounds have been demonstrated to be agents with antioxidant, anticancer, anti-inflammation, and antimicrobial effects [9,10,11,12]. In addition, due to the low cytotoxicity of these compounds, researchers have shown considerable interest in the potential health benefits of natural polyphenols and development of products manufactured from fruits or foods with substantial amounts of polyphenols to explore additional applications in human health and medicinal therapies [13,14,15,16].

Currently, microbial drug resistance has become a crucial global concern. Antibiotic overuse has resulted in the emergence of a drug-resistant strain of *S. aureus*, namely MRSA, leading to several challenges in clinical therapy. The major mechanisms underlying drug resistance include enzymatic modification, mutation of the antibiotic target, and microbial drug efflux via the efflux pump [17]. The prognosis of MRSA infection is worse than that of infections caused by other susceptible organisms, including the risk of treatment failure, relapse, and high mortality [6]. Additional investigations are therefore required to evaluate the benefits of new antimicrobial molecules against MRSA and to develop more effective therapeutic strategies. Although considerable research in recent years has focused on the potential antimicrobial properties of polyphenols, limited attention has been paid to address the problem of microbial resistance. Thus, in this study, we investigated the antimicrobial effects of TGII, a natural pure compound, against *S. aureus* isolates. We evaluated whether TGII inhibits the growth of MSSA and MRSA and further investigated how the drug-resistant mechanism of MRSA is regulated by TGII.

## 2. Results

### 2.1. TGII Exhibits Potent Antimicrobial Effects Against S. Aureus Strains

**Figure 1:**
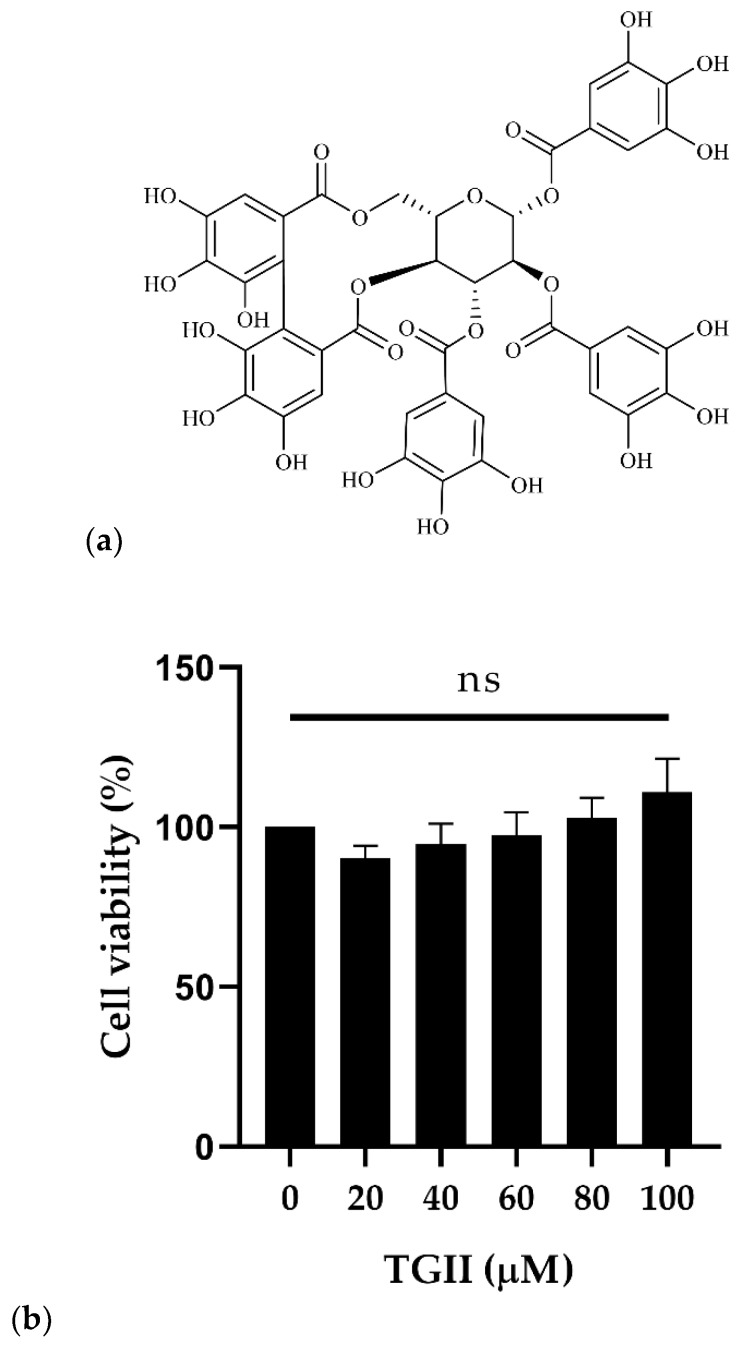
Effects of Tellimagrandin II (TGII) on cell viability in peripheral blood mononuclear cells (PBMCs). (**a**) Chemical structure of Tellimagrandin II; (**b**) PBMCs were treated with TGII at a dose ranging from 0 to 100 μM for 24 h, and the viability of the treated cells was determined using the WST-1 assay (quantitative data were measured at least three times and are presented as mean ± SD).

**Figure 2:**
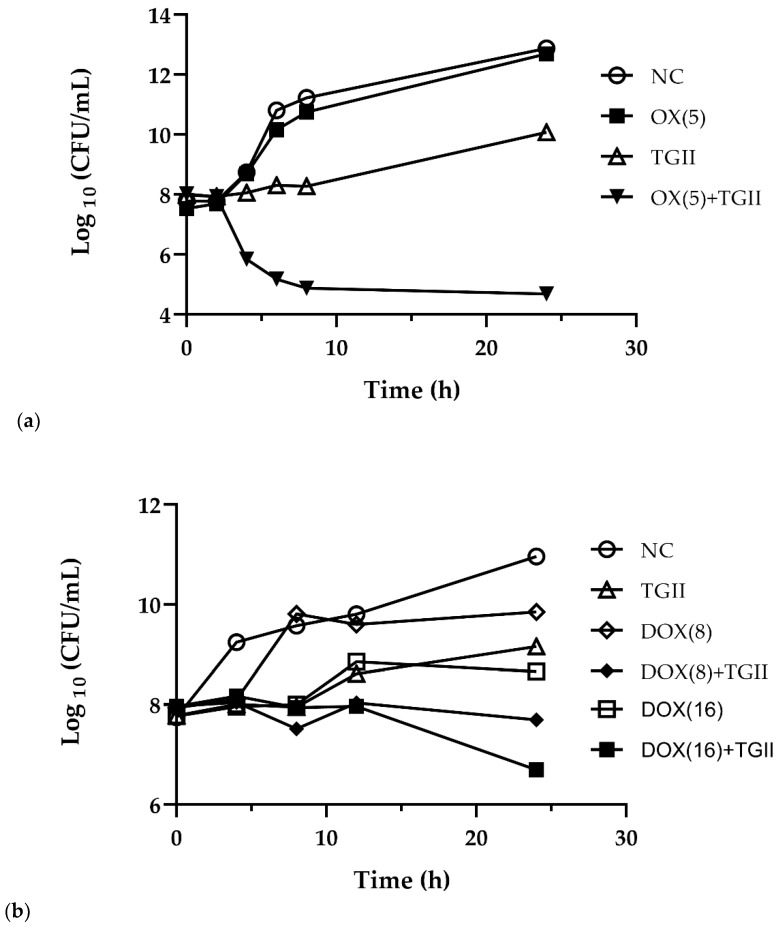
Time-kill kinetics of TGII, (**a**) oxacillin (OX), (**b**) doxycycline (DOX), and TGII combined with OX or DOX against clinically isolated MRSA strain (MRSA 19615). The treatment conditions are represented by the different symbols. NC: negative control; TGII: 40 μg/mL; OX(5): 5 μg/mL; DOX(8): 8 μg/mL; DOX(16): 16 μg/mL. These experiments were repeated at least three times.

Figure 1a shows the chemical structure of TGII (molecular formula: C_41_H_30_O_26_; molecular weight: 938.7 g/mol) [18]. First, we used the WST-1 assay to evaluate the cytotoxicity of TGII toward human cells. As seen in Figure 2a, the highest TGII dose of 100 μM (93.9 μg/mL) with an exposure time of 24 h had no cytotoxicity toward human peripheral blood mononuclear cells (PBMCs). Next, we estimated the antistaphylococcal activity of TGII. Table 1 presents the minimum inhibitory concentrations (MICs) of TGII against the indicated *S. aureus* strains. The clinically isolated MSSA was sensitive to treatment with conventional antibiotics such as oxacillin, erythromycin, and doxycycline. By contrast, MRSA resisted almost all antibiotics, and we found the resistance of the MRSA33591 strain to levofloxacin and doxycycline. Notably, the MICs of TGII for MSSA and MRSA were 64 and 128 μg/mL, respectively (Table 1). These results revealed that TGII exerted significant antibacterial activity against both MSSA and MRSA. Moreover, compared with the antibiotics listed in Table 1, TGII exhibited potent antibacterial activity against all the *S. aureus* strains.

To establish whether a synergistic or an additive relationship existed between antibiotics and TGII, we used clinically isolated MSSA and MRSA strains to assess fractional inhibitory concentration (FIC) values of TGII combined with conventional antibiotics. Table 2 shows that all the FICs ranged from 0.008 to 0.063, indicating the additive or the synergistic effects of the combination of TGII and oxacillin against clinically isolated MRSA. Similar results are presented in Table 3; the FIC values indicate that TGII combined with antibiotics such as oxacillin and doxycycline exhibited synergistic antibacterial activity against MRSA. These results indicate that combination treatment with TGII could effectively improve drug resistance in *S. aureus* isolates. Notably, compared with the minimal inhibitory concentration (MIC) values of antibiotics alone, the MIC values of the combination of TGII and oxacillin, ampicillin, erythromycin, or doxycycline indicated more notable antibacterial effects against MSSA (Table 3). Altogether, the present results suggest that TGII alone exerts antistaphylococcal activity, and TGII in combination with low-dose antibiotics displays synergistic effects against MSSA and MRSA; this allows for the reduction of the effective dose of current antibiotics, thereby overcoming the predicament of drug resistance in *S. aureus* isolates.

### 2.2. TGII Possesses Potent Bactericidal Activity Against Drug-Resistant S. Aureus Strains

The MIC values provided preliminary confirmation of the antibacterial activity of TGII against MRSA. We next performed the time-kill kinetic study to evaluate the bactericidal activity of TGII against MRSA. As shown in Figure 2a, we found obvious reduction in the cfu count (~3.0 log_10_ CFU/mL) for the TGII-treated group compared with the control or the oxacillin-treated group within the early 8 h of incubation. Consistently, TGII combined with low-dose oxacillin exerted a more significant reduction in the cfu count (~6.0 log_10_ CFU/mL) of MRSA at the early stage. Notably, the observation of the long-term bactericidal activity revealed that TGII exerted excellent synergistic effects in combination with oxacillin, which caused a more than two-fold significant reduction in the MRSA cfu count (~8.0 log_10_ CFU/mL) after 24 h incubation. As seen in Figure 2b, MRSA treated with TGII combined with another resistant antibiotic, doxycycline, also showed a decreased cfu of ~2.0 log_10_ (CFU/mL) at 24 h from the beginning of the observation period. These results indicate that TGII exerts bactericidal activity against MRSA. Moreover, when MRSA was treated with TGII combined with oxacillin or doxycycline, the bactericidal effect was displayed not only in the early incubation period but also in the bacterial regrowth stage during long-term incubation.

### 2.3. TGII Reduces the Resistance of MRSA by Regulating PBP2a Expression

**Figure 3:**
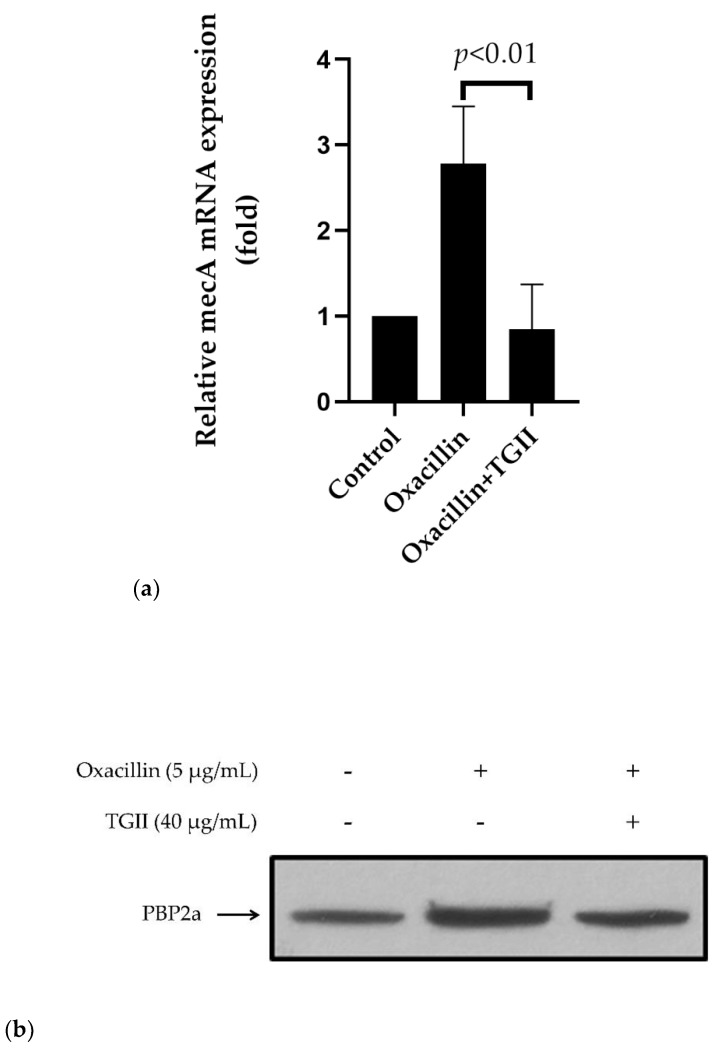

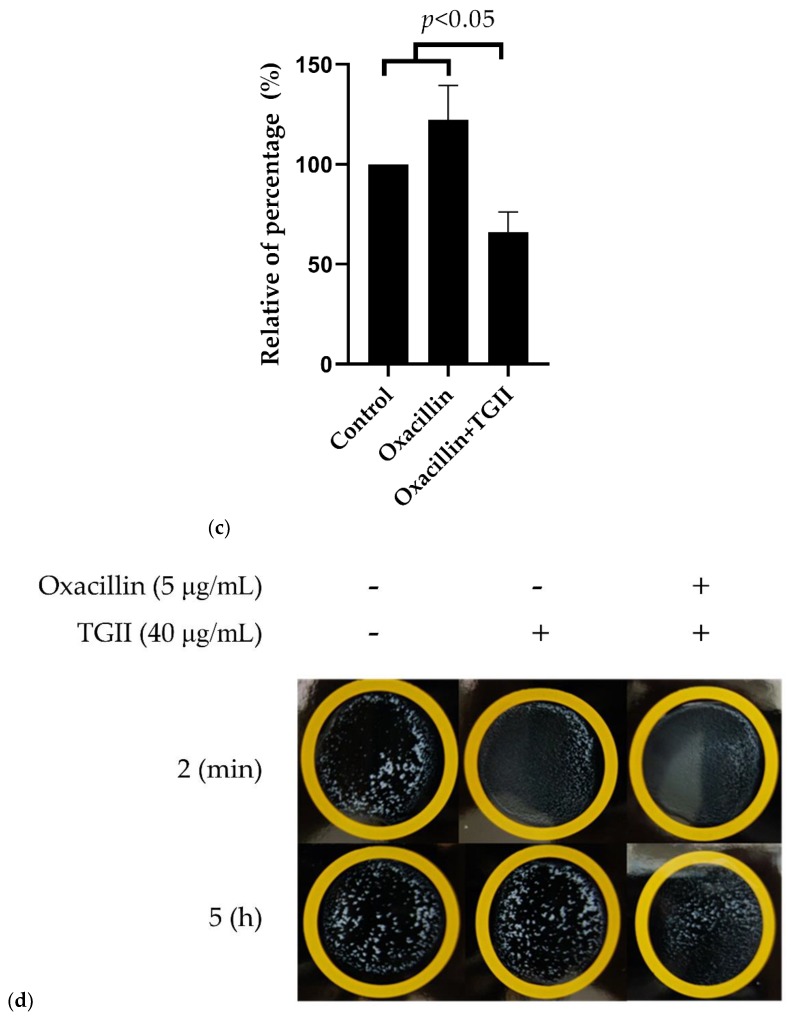

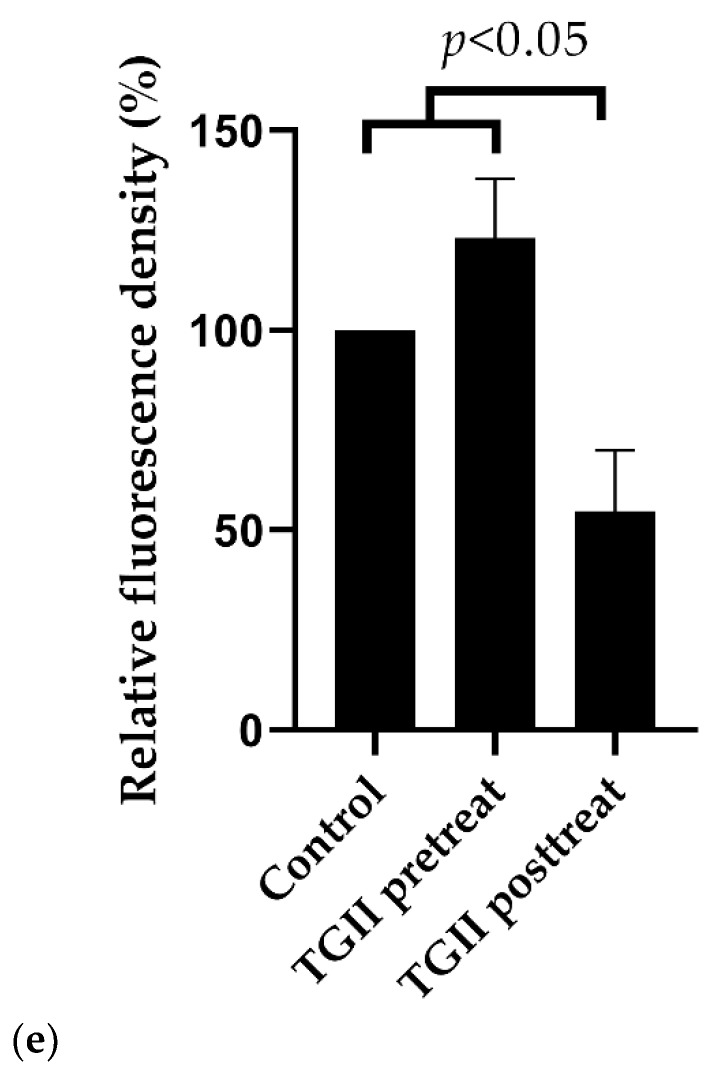
TGII regulates the expression and the penicillin-binding ability of penicillin-binding protein 2a (PBP2a) in the MSRA strain. The clinically isolated MRSA was treated with oxacillin alone or oxacillin in combination with TGII by following the procedures described in the Materials and Methods section. The present data (shown as mean ± SD, n = 3) reveal the following: (**a**) mecA mRNA expression determined using qPCR; (**b**) PBP2a expression determined using Western blot analysis; and (**c**) the quantitative graph of Western blot from (**b**); MRSA treated with oxacillin or oxacillin combined TGII were normalized to control cells, respectively; (**d**) PBP2a latex agglutination test; (**e**) the relative fluorescence density of BOCILLIN FL; the protein lysates were incubated with BOCILLIN FL for 30 min according to the following conditions: control (without TGII treatment), TGII pretreat (TGII treatment before protein extraction), and TGII posttreat (TGII treatment after protein extraction).

Among the drug resistance mechanisms in *S. aureus*, the emergence of penicillin-binding protein 2a (PBP2a) is the major type of β-lactam mutation in MRSA strains. This novel target protein is encoded from the mecA gene, enabling *S. aureus* to resist antibiotics [19,20]. To further elucidate the mechanism of the anti-MRSA activity of TGII, we determined the expression of the mecA-PBP2a cascade of MRSA after TGII treatment. First, we observed whether TGII regulated the messenger RNA products of the mecA gene. Figure 3a shows that treatment with TGII combined with oxacillin drastically reduced the levels of mecA mRNA in the MRSA strain. As expected, the level of PBP2a, the mecA-encoded protein, significantly declined after TGII treatment (Figure 3b–c). In addition, we used the latex agglutination assay to detect the level of PBP2a in the MRSA strain. Compared with the strains treated with TGII alone, the MRSA strains exposed to TGII combined with oxacillin for 5 h showed an obvious reduction in agglutination (Figure 3d). To further clarify whether TGII alters the penicillin-binding activity of PBP2a in MRSA strains, we sonicated and extracted the total protein from the MRSA strain under treatment, as described in the Materials and Methods section, followed by incubation with BOCILLIN FL penicillin, and we then detected the fluorescence after SDS-PAGE electrophoresis. As shown in Figure 3e, compared with the control group, the penicillin-binding ability of PBP2a significantly declined in the MRSA strain treated with TGII before protein lysate extraction (represented as TGII pretreat in Figure 3e). However, the improvement in penicillin-binding capacity was reversed on incubation of protein lysate with TGII after harvesting (represented as TGII posttreatment in Figure 3e). Overall, TGII treatment not only resulted in a significant reduction of both mecA mRNA (*p* < 0.05) and PBP2a protein (*p* < 0.05) in MRSA strains but also altered the penicillin-binding ability of PBP2a. These results indicate that TGII can overcome the drug-resistant mechanism originating from the PBP mutation in resistant *S. aureus* strains.

### 2.4. TGII Disrupts the Cell Wall Integrity of MRSA

**Figure 4:**
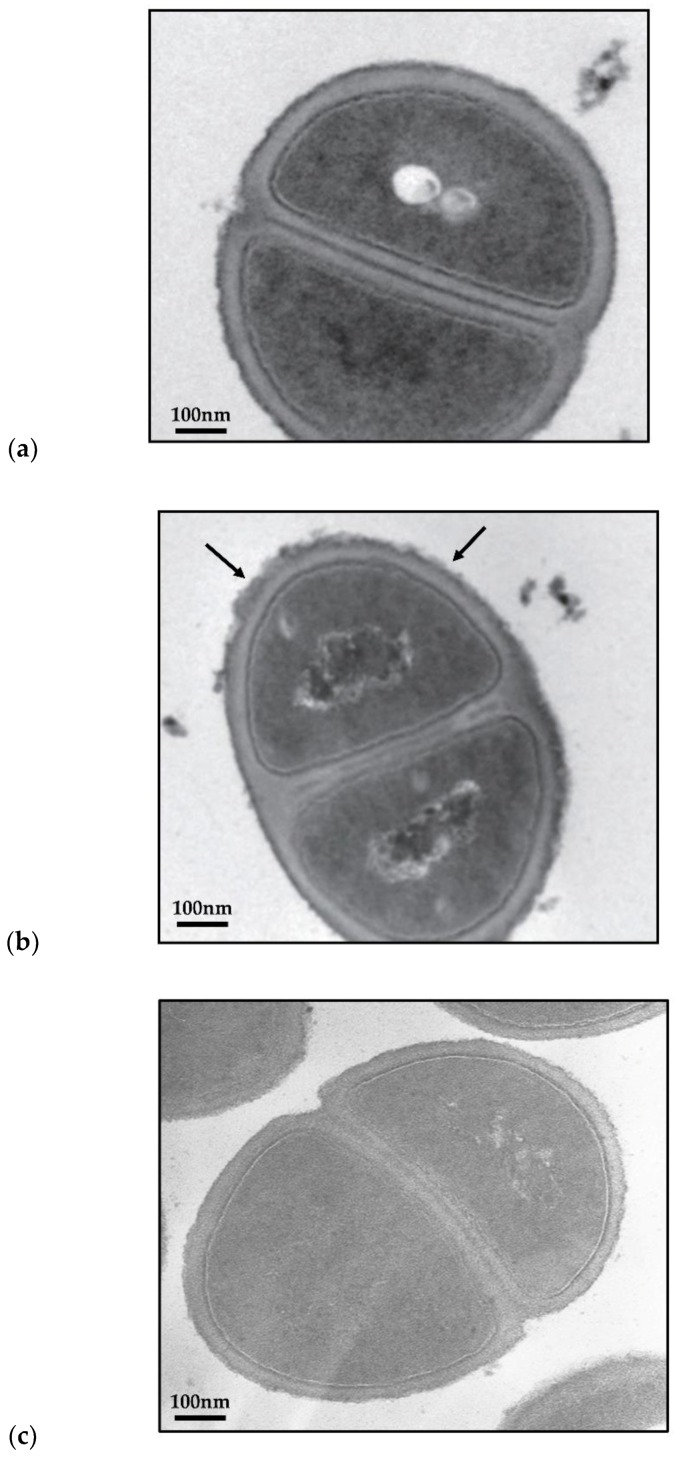

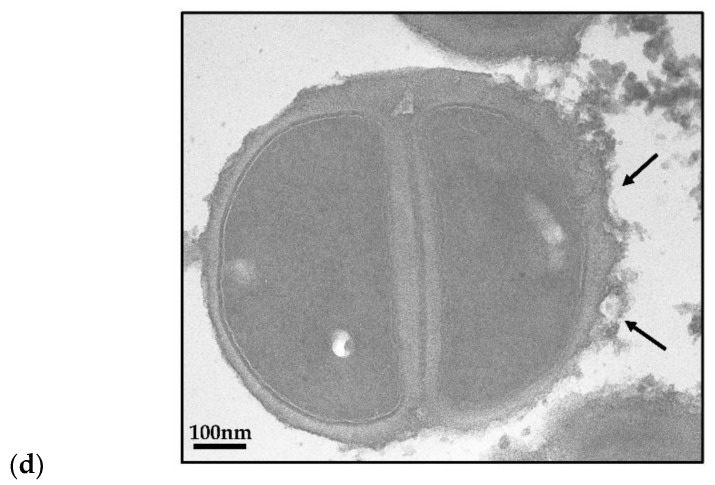
TEM micrographs of MRSA incubated at the following conditions: (**a**) control; (**b**) 40 μg/mL TGII for 10 min; and (**c**) control, (**d**) 40 μg/mL TGII for 24 h. The arrows in Figure 4b,d indicate cell wall alteration in MRSA cells. Untreated MRSA cells served as the control group in the TEM imaging observation.

To further evaluate whether the integrity of the cell wall of MRSA can be disrupted by TGII treatment, we performed a TEM characterization of MRSA incubated with TGII to observe the cell wall morphology. Figure 4 shows the TEM micrograph of the MRSA strain treated with 40 μg/mL TGII over 10 min and 24 h. The images reveal the initiation of the disruption of the cell wall of the TGII-treated MRSA strains at the first 10 min incubation (Figure 4b); by contrast, the entire smooth morphology was seen in the control cells (untreated MRSA cells, see Figure 4a). After 24 h of incubation, TGII further disrupted the cell membrane more severely and caused the loss of internal cell content (Figure 4d) compared with control cells (Figure 4c). This direct evidence suggests that the anti-MRSA capability of TGII starts with the disruption of the bacterial cell wall followed by altering the integrity of the bacterial membrane.

## 3. Discussion

Microbes may acquire resistance to conventional antibiotics through de novo mutations or by receiving resistance genes from other organisms [21,22]. Consequently, bacteria develop several adaptive mechanisms to eliminate the effects of antibacterial agents, including inactivating or excluding a drug and a structure conformation modification for bypassing the drug action [23]. Notably, the global spread and the increasing populations of antibiotic-resistant microbes are global public health concerns [4,24]. More than two million people are infected by antibiotic-resistant pathogens worldwide, resulting in over 700,000 deaths each year [24]. Developing strategies to overcome the predicament of drug-resistant *S. aureus* isolates is essential. Studies have documented that the frequency of MRSA in developed countries in Asia (e.g., Japan and Korea) ranges from 0% to approximately 70% and 40% in the United Kingdom and the United States. In addition to these developed countries, the epidemic trends of MRSA is worse in developing countries, and the prevalence of MRSA infections has rapidly increased in the last decade [25]. Mutation-triggered resistance in *S. aureus* has been rapidly occurring; thus, the development of new anti-MRSA agents is urgent and essential. Otherwise, several plant-derived phytochemicals have been reported to display satisfactory antibacterial activity, especially against resistant strains. [26,27,28,29,30]. To process the invaded microbes, plants have developed multiple defense strategies, including phytoanticipins (direct inducible chemical defense) and phytoalexins (gene-level inducible chemical defense) and other constitutive chemical defense mechanisms. These mechanisms enable the plants to acquire a series of structurally diverse secondary metabolites [31,32]. Therefore, we aimed to identify local plants that could potentiate the effects of conventional antibiotics.

For this purpose, in this study, we estimated whether TGII can overcome the resistance of *S. aureus* to conventional antibiotics. The antibacterial capacity of this plant polyphenol compound alone or in combination with commercial antibiotics against MSSA and MRSA strains and the underlying mechanisms was evaluated in the present study.

Our data revealed that the MIC of TGII was 64–128 μg/mL against susceptible and resistant *S. aureus*, which is drastically lower than the MIC values of reference antibiotics, and TGII exerted growth inhibition without significant cytotoxicity (Table 1 and Figure 2). Further, the FIC values strongly evidence that TGII in combination with resistant antibiotics exhibited synergistic antibacterial activity against MRSA. Notably, compared with treatment with oxacillin alone, oxacillin combined with TGII obviously reduced the MIC from 512 to 2 μg/mL in the MRSA ATCC33591 strain, whereas the FIC (0.004) value represents a significant synergistic effect (Table 2 and Table 3). The effective antimicrobial ability of TGII suggests that the MRSA strain was as sensitive as the MSSA strain after TGII treatment.

TGII was extracted from the shells of *Trapa bispinosa*, which is a type of economic agriculture product in southern Taiwan and is locally known as water chestnut. The fruit and the seeds of *Trapa bispinosa* have been well established as nutrition-rich foods with beneficial medical properties; however, the bioactivity of the component from shells remains unclear [7]. To our knowledge, this is the first study to estimate the biofunction of a major compound from the shells of *Trapa bispinosa*. In relation to our findings, Yamaguchi et al. reported that TGII exhibits a potent inhibitory effect against fungus with an MIC of 1.6 μM. In addition, TGII acts synergistically with nystatin, amphotericin, and fluconazole and causes morphological alterations in yeast cells [33]. Furthermore, TGII has been proven to exert hepatoprotective properties by reducing the alanine aminotransferase (ALT) levels by up to 45% and the aspartate aminotransferase (AST) levels by up to 45% in vivo [34]. We demonstrated that TGII exerts bactericidal activity against drug-resistant *S. aureus* strains. In this study, we provided similar findings that TGII alone or in combination with other resistant antibiotics plays a crucial role in the bactericidal activity not only in the early incubation period but also in the bacterial regrowth stage during long-term incubation (Figure 2). Moreover, TGII interfered with the synthesis and the function of resistance-derived proteins in MRSA (i.e., inhibition of *mecA* transcription and reduction of the penicillin-binding capacity of PBP2a) by regulating the de novo mutation mechanisms of MRSA (Figure 3). In addition, consistent with previous reports [35,36], the bacterial morphology was severely disrupted with TGII treatment, and significant cell plasmolysis was observed. The results presented in Figure 4 indicate that TGII caused extensive damage to MRSA, which encourages further investigation of TGII in the near future. Furthermore, herein we highlight some future research directions. First, we demonstrated the bactericidal activity of TGII only against MSSA and MRSA strains, which are gram-positive bacteria. This evidence is not generalizable to the overall antimicrobial effects obtained from TGII. Therefore, to more thoroughly define the phytochemical benefits of TGII, the bioactivities of TGII toward other microbial infections must be further investigated (i.e., gram-negative bacteria and virus). Second, biofilm formation is the critical step for *S. aureus* adhesion [37]; therefore, the regulation effect of TGII in biofilm formation or other virulence factors must be further estimated in future works.

Recently, the emergence of the multidrug-resistant bacteria has raised global alarm. Because the therapeutic strategies for infected patients are extremely limited currently, identifying new candidates of antimicrobial agents is urgently required. Altogether, TGII inhibits MRSA by altering the bacterial morphology and negatively regulating PBP2a-mediated β-lactam resistance. These findings suggest that TGII is a novel antistaphylococcal agent due to its potent effectiveness in inhibiting the growth of MSSA and MRSA strains.

## 4. Materials and Methods

### 4.1. Materials

TGII was provided and identified by Lih-Geeng Chen (NCU, Jiayi, Taiwan). The bio-activity of anti-bacterial infection obtained from TGII was granted to the United States patent on 10 March 2015 (Patent No. US8,975,234 B2). The abstract and the detail claim can be seen in the open patent document. All the conventional antibiotics were purchased from Sigma-Aldrich (St Louis, MO, USA), namely, oxacillin, erythromycin, ampicillin, kanamycin, levofloxacin, vancomycin, and doxycycline, as well as BOCILLIN FL used to detect the penicillin-binding capacity of PBP2a. Mueller–Hinton (MH) broth for bacteria dilution and enrichment culture was purchased from BD Company (San Diego, CA, USA). The PBP2a latex agglutination assay kit was purchased from Denka Seiken Co., Ltd. (Tokyo, Japan).

### 4.2. Bacterial Strains and PBMCs

The clinical MSSA and MSSA isolates were provided by the clinical microbiology laboratory at Kaohsiung Medical University Hospital and were used as received. The reference MRSA strain (ATCC 33591) was purchased from Biosource Collection and Research Center (Taiwan) and subcultured on MH agar.

PBMCs were obtained from three healthy donors under the IRB-approved protocol. The vein blood samples were first centrifuged at 2000 rpm for 15 min at room temperature. After plasma removal, equal amounts of the discarded plasma were refilled using sterile phosphate buffered saline (PBS), and the cells were centrifuged at 1000 rpm for 10 min. Then, a layer of white blood cells plus some red blood cells was transferred to the tube using Ficoll-Hypaque. Finally, we obtained the buffy coat layer after centrifugation at 2000 rpm for 30 min, which was cultured in RPMI 1640 containing 10% fetal bovine serum (FBS).

### 4.3. Cell Viability Determination

The obtained PBMCs were plated in 96-well plates at a density of 6000 cells/well. After 24 h incubation, the cells were treated with TGII at a dose ranging from 0 to 100 μM for 24 h. Based on the 1/10 ratio, 10 mL of WST-1 reagent was then added to each well. Finally, absorbance was determined using an ELISA reader at a test wavelength of 450 nm and a reference wavelength of 630 nm. In addition, DMSO was used as a solvent to resolve TGII at final concentrations in the culture solution, which served as the toxin control in the cell viability assay.

### 4.4. MIC Assay

Based on a previous study, MIC values of TGII were determined for all *S. aureus* strains by using the twofold broth serial dilution method [4]. Briefly, *S. aureus* was diluted in MH broth and cultured overnight to achieve optimal cell density of nearly 2 × 10^6^ cfu/mL (OD_600_ = 0.01). Then, the cells were treated with several dilutions of TGII or commercial antibiotics ranging from 2 to 512 μg/mL in MH broth and then incubated at 37 °C. After 24 h incubation, MICs were determined by detecting OD600 absorbance values corresponding to the minimal concentration of the compounds that caused complete inhibition of visible growth of bacteria.

### 4.5. FIC Determination

To evaluate the synergistic, the antagonistic, or the additive effects of TGII and conventional antibiotics, FIC values were determined according to the following formulas [25]:

FIC value = MIC of antibiotics combined with TGII/MIC of antibiotics alone.

Based on the definition, the effects of combined agents were determined to be synergistic (FIC ≤ 0.5), additive (0.5 < FIC < 1), indifferent (1 < FIC < 2), or antagonist (FIC ≥ 2) [38].

### 4.6. Time-Kill Curve Determination

According to a previous study [39], time-kill curves were used to evaluate the bactericidal effect of TGII alone or TGII combined with antibiotics against *S. aureus*. Briefly, all *S. aureus* strains were cultured overnight in MH broth, and the turbidity equivalent was then adjusted to a 0.5 Mcfarland standard. The primary inoculum (~5 × 10^5^ cfu/mL) was prepared and aliquoted to different tubes for treatment with TGII or TGII combined with antibiotics at concentrations, as described in Figure 2. The cultures were then incubated at 37 °C, and 50 μL of the cultures were plated onto MH agar plates at 0, 2, 4, 6, 8, and 24 h of incubation. Finally, cfu counts were determined after overnight incubation. A significant bactericidal effect was defined as a more than 3 log_10_ (CFU/mL) reduction in each primary inoculum at any time of incubation.

### 4.7. Quantitative Real-Time PCR

The RNA material was extracted from *S. aureus* by using the high pure RNA isolation kit purchased from Roche Molecular Systems Inc (Pleasanton, CA, USA) and was reverse-transcribed to cDNA using the SuperScript III Reverse Transcriptase kit purchased from Thermo Fisher Scientific Inc (Waltham, MA, USA); both procedures were performed according to the manufacturer’s instructions. Based on a previously described method [40,41], real-time quantitative PCR (qPCR) analysis was then performed using the Roche LightCycler (Mannheim, Germany) to evaluate the target mRNA levels. We used the ΔΔ^C^t method to calculate the target gene expression. All data presented in qPCR results were normalized to *ftsZ*, the housekeeping gene for *S. aureus*. Primer sequences used for qPCR in the study are listed in Table 4 [42].

### 4.8. Western Blot Analysis

After treatment of TGII or TGII combined oxacillin, MRSA was then incubated at 37 °C for 24 h. Then, cells were sonicated and centrifuged at 15,000 rpm for 30 min. After that, protein lysates were harvested and determined the total protein concentration by the BCA kit. An equal amount (30 μg) was resolved by 10–12% SDS-PAGE and then transferred onto PVDF membrane. After blocking in TBST buffer containing 2% bovine serum albumin (BSA), the membrane was probed with the specific antibody.

### 4.9. PBP2a Latex Agglutination Assay

According to the manufacturer’s instructions and based on a previous study [43], we performed a rapid test to screen the penicillin-binding capacity of PBP2a of *S. aureus*. Briefly, four drops of the extraction reagent provided in the commercial kit were used to suspend a 1 μL loop of bacterial cells. Then, the suspension was boiled for 3 min. After cooling to room temperature, one drop of extraction reagent 2 was added to the sample followed by thorough mixing using a vortex mixer. Then, the suspension was centrifuged at 1500 × g for 5 min. The supernatant (50 μL) was obtained and mixed with one drop of anti-PBP2a monoclonal antibody-sensitized latex beads. Finally, the samples were gently shaken for 3 min prior to the visible assessment of agglutination. In addition, one drop of negative control latex provided in the kit was mixed with 50 μL of the supernatant, representing the negative control.

### 4.10. BOCILLIN FL Assay

To evaluate the regulation of MRSA PBP2b by oxacillin or the antibiotic and TGII combination, we performed SDS-PAGE-based concentration response experiments by using the fluorescent penicillin BOCILLIN FL as the reporter molecule. According to the manufacturer’s instructions and based on a prior study [44], we sonicated and obtained protein extracts from the treated MRSA. Then, BOCILLIN FL was added to each protein lysate in a final reaction volume of 35 μL. The mixture was incubated at 32 °C for 30 min prior to SDS-PAGE electrophoresis. Finally, the gels were imaged under UV light followed by densitometry analysis.

### 4.11. Transmission Electron Microscopy (TEM)

Briefly, we followed the previous study to obtain MRSA exponential-phase cultures by overnight incubation in MH broth at 37 °C until they reached the mid-logarithmic phase of growth [45]. Subsequently, the exponential-phase MRSA cultures were treated with TGII at the MIC dose (40 μg/mL) for 10 min or 24 h. After treatment, cell pellets were obtained after centrifugation (10,000 × g for 10 min) and fixed with 2.5% glutaraldehyde and 2.5% formaldehyde for 2 h. Finally, ultra-thin sections were prepared and visualized by electron microscopy (JEM-1200EX, JEOL, Tokyo, Japan).

### 4.12. Statistical Analysis

All data in the study are presented as mean ± standard deviation (SD), with n indicating the number of experiments, after which they were analyzed using Student’s *t* test. All differences were considered significant at a *p* value of <0.05.

## 5. Conclusions

In conclusion, we evidenced the antibacterial effects of TGII against MRSA, which enables the effective dose of current antibiotics to be reduced and the predicament of drug-resistant *S. aureus* isolates to be overcome.

## Figures and Tables

**Table 1:** Minimal inhibitory concentration (MIC) values of methicillin-sensitive *S. aureus* (MSSA) and methicillin-resistant *S. aureus* (MRSA) after treatment with Tellimagrandin II and antibiotics.

Antibiotics/TGII	MIC of MSSA ^1^ (μg/mL)	MIC of MRSA ^2^ (μg/mL)			
		19615	18631	18271	33591
Oxacillin	4	512	> 512	> 512	> 512
Ampicillin	> 512	> 512	> 512	> 512	> 512
Erythromycin	128	> 512	> 512	> 512	> 512
Kanamycin	> 512	> 512	> 512	> 512	> 512
Levofloxacin	> 512	> 512	> 512	> 512	32
Doxycycline	128	> 512	256	> 512	64
TGII	64	128	128	128	128

^1^ MSSA is a clinical isolate; ^2^ MRSA 19615/18631/18271 are clinical isolates; MRSA 33591 is the ATCC strain.

**Table 2:** MIC and fractional inhibitory concentration (FIC) of oxacillin with or without TGII against clinically isolated MRSA strains.

MRSA Strain	MIC of Oxacillin (μg/mL)		FIC	Combination Effect
	TGII ^1^ (−)	TGII (+)		
MRSA1 ^2^	512	4	0.008	Synergy
MRSA2	512	4	0.008	Synergy
MRSA3	512	4	0.008	Synergy
MRSA4	512	2	0.004	Synergy
MRSA5	512	2	0.004	Synergy
MRSA6	256	2	0.008	Synergy
MRSA7	256	2	0.008	Synergy
MRSA8	128	2	0.016	Synergy
MRSA9	128	2	0.016	Synergy
MRSA10	64	2	0.031	Synergy
MRSA11	64	4	0.063	Additive
MRSA12	64	4	0.063	Additive
MRSA13	64	2	0.031	Synergy
MRSA14	32	2	0.063	Additive

^1^ The administrated dose of TGII is 40 μg/mL. ^2^ Strain number listed in the table depicts the disconnect symbols of clinical isolates.

**Table 3:** MIC and FIC of the conventional antibiotics with or without TGII against MSSA and MRSA.

Antibiotics	TGII ^1^	MSSA ^2^		MRSA ^2^							
				19615		18631		18271		33591	
		MIC ^3^	FIC	MIC	FIC	MIC	FIC	MIC	FIC	MIC	FIC
Oxacillin	−	4	−	512	−	512	−	512	−	512	−
	+	2	0.5	4	0.008	8	0.02	16	0.03	2	0.004
Ampicillin	−	512	−	512	−	512	−	512	−	512	−
	+	256	0.50	512	1.00	512	1.00	16	0.031	512	1.00
Vancomycin	−	32	−	16	−	16	−	16	−	4	−
	+	32	1.00	32	2.00	32	2.00	32	2.000	4	1.00
Levofloxacin	−	512	−	512	−	256	−	512	−	512	−
	+	512	1.00	512	1.00	512	2.00	512	1.00	512	1.00
Erythromycin	−	128	−	512	−	512	−	512	−	512	−
	+	64	0.50	512	1.00	512	1.00	512	1.00	512	1.00
Kanamycin	−	512	−	512	−	512	−	512	−	512	−
	+	512	1.00	512	1.00	512	1.00	512	1.00	512	1.00
Doxycycline	−	128	−	512	−	256	−	512	−	64	−
	+	8	0.063	8	0.016	2	0.008	2	0.004	64	1.00

^1^ The administrated dose of TGII is 40 μg/mL; the symbols (+, −) are depicted with or without TGII treatment. ^2^ Strains definition described in Table 1; ^3^ MIC values are represented in μg/mL.

**Table 4:** Primer sequences used for qPCR in the present study.

Target Gene	Product Length (bp)	Sequence	
*mecA*	94	Forward	CTGCTATCCACCCTCAAACAG
		Reverse	TCTTCGTTACTCATGCCATACA
*ftsZ (S. aureus)*	217	Forward	TTACTGGTGGCGAGTCATTG
		Reverse	TTTACGCTTGTTCCGAATCC

## Abbreviations

TGII	Tellimagrandin II
TEM	transmission electron microscopy
MRSA	methicillin-resistant *Staphylococcus aureus*
MSSA	methicillin-sensitive *Staphylococcus aureus*
PBP2a	penicillin-binding protein 2a
PBMCs	peripheral blood mononuclear cells
MH	Mueller-Hinton
MIC	minimal inhibitory concentration
FIC	fractional inhibitory concentration
